# Prognostic Impact of Serum CRP Level in Head and Neck Squamous Cell Carcinoma

**DOI:** 10.3389/fonc.2022.889844

**Published:** 2022-06-29

**Authors:** Yaoting Zhang, Dongsheng Gu

**Affiliations:** Department of Otorhinolaryngology-Head and Neck Surgery, The Affiliated Huaian No. 1 People’s Hospital of Nanjing Medical University, Huai’an, China

**Keywords:** biomarker, prognosis, head and neck squamous cell carcinoma, CRP, C-reactive protein

## Abstract

**Objective:**

This study evaluated the association of pretreatment serum C-reactive protein (CRP) level with prognosis in patients with head and neck squamous cell carcinoma (HNSCC).

**Methods:**

Within a single-center retrospective study, HNSCC patients receiving treatment between 2014 and 2016 were analyzed regarding the prognostic value of CRP serum levels. X-Tile software was used to determine the optimal cutoff value of serum CRP level. The log-rank test and Kaplan–Meier method were used to assess the effects of CRP level on prognosis in patients with HNSCC. Univariate and multivariate analyses (enter method) using a Cox proportional hazards model were utilized to identify prognostic indicators of progression-free survival (PFS) as the primary outcome and overall survival (OS) as the secondary outcome.

**Results:**

A total of 221 patients with HNSCC were assessed for eligibility, and 208 cases were included in the analysis. The HNSCC patients in the low-group (CRP ≤11.3 mg/L) showed better survival than those in the high-group (CRP > 11.3 mg/L). The univariate and multivariate analyses showed that N1-3 stage and a high serum CRP level (>11.3 mg/L) were unfavorable prognostic factors for PFS and OS in patients with HNSCC.

**Conclusion:**

Serum CRP level is an independent prognostic marker for patients with HNSCC. CRP level could be regarded as a novel prognostic factor for HNSCC patients.

## Introduction

Head and neck cancer is the sixth most common cancer worldwide, and more than 600,000 new cases are diagnosed annually (1). Head and neck squamous cell carcinomas (HNSCCs) are the most common type of head and neck tumor worldwide and represent approximately 95% of all head and neck cancers. Tobacco exposure, alcohol consumption, and human papillomavirus (HPV) infection are known risk factors for HNSCC (2, 3). The therapeutic options for patients with HNSCC include surgery, chemotherapy, radiation, and immunotherapy (4–6). However, little progress has been made in the treatment of HNSCC, with a 5-year overall survival (OS) rate of ~50% (1), due to a propensity for locoregional recurrence and distant metastases (3). Therefore, it is necessary to identify effective predictive and prognostic biomarkers that may help to improve the prognosis of HNSCC.

A number of novel prognostic biomarkers for HNSCC have been explored (7–9). However, none of these has been recommended for routine clinical testing. A number of blood markers are routinely measured before and during routine clinical treatments, including C-reactive protein (CRP). Cancer-related systemic inflammation has been regarded as a pivotal determinant of disease progression and survival in most cancers (10–13); CRP is among these inflammatory markers. Several recent studies investigated the association between serum CRP level and HNSCC patient survival, but they yielded conflicting results (14–29). It is unclear whether serum CRP levels can be used to predict the survival of Han Chinese patients with HNSCC. Therefore, this study examined the role of serum CRP in the prognosis of patients with HNSCC.

## Patients and Methods

### Patients

All patients with HNSCC admitted to our hospital between May 2014 and April 2016 were screened for inclusion in this retrospective study according to the following eligibility criteria: diagnosis based on histological evidence, age >18 years, and treatment with surgery, chemotherapy, or radiation therapy. The exclusion criteria were patients with a family history of HNSCC, patients with other cancers, patients with infectious or autoimmune diseases, and patients without any treatment. Clinical information, including age, sex, smoking status, alcohol drinking status, UICC stage, tumor localization, and HPV infection status, was recorded. Smokers were defined as follows: individuals smoked over two cigarettes a day for at least 1 year. Drinkers were defined as those whose alcohol intake per day was more than 20 grams for at least 1 year. HPV-infected individuals were defined as to who were infected with HPV 16 or other high-risk HPV genotypes. HPV16 status was not used as a surrogate marker for HPV status. All tumors were analyzed regarding HPV DNA with PCRs. The serum CRP level was obtained from the medical records of all patients prior to treatment, which was measured among all patients on the first day after admission. Healthy subjects receiving health checkups at the same time were included as controls. Subjects with cancers or infectious or autoimmune diseases were excluded from the control group. This study was approved by the Ethics Committee of our hospital and was performed in accordance with the Declaration of Helsinki.

### Follow-Up

The main outcome was progression-free survival (PFS), defined as the time from initial diagnosis to disease progression or death from any cause. The secondary outcome was OS, which was defined as the time from initial diagnosis to death from any cause. The patients were followed up once a month after treatment for the first 2 years and once 3 months for the following years. The follow-up time ended in November 2021.

### Statistical Analysis

Using the minimal *P*-value approach, X-tile software (ver. 3.6.1; Yale University School of Medicine, New Haven, CT, USA) was used to determine the cutoff value of serum CRP level for analyzing the survival analysis (30). The patients with HNSCC were divided into two groups (low-CRP group and high-CRP group) according to the CRP cutoff level. In terms of serum CRP levels, the comparison between HNSCC patients and healthy controls was analyzed by *t*-test (normally distributed variables) or Mann–Whitney *U*-test (non-normally distributed variables). We utilized the chi-square test or Fisher’s exact test to compare the patient characteristics between the low-CRP group and high-CRP group. PFS and OS were determined using the Kaplan–Meier method. The significance of differences was evaluated using the log-rank test. A Cox proportional hazards model was utilized to calculate the hazard ratio (HR) and corresponding 95% confidence interval (CI). Univariate and multivariate Cox regression analyses (enter method) were performed to identify factors affecting the prognosis of HNSCC. Statistical significance was defined as a *P*-value lower than 0.05. Statistical analyses were performed using IBM SPSS Statistics (version 21.0; IBM Corp., Armonk, NY, USA), GraphPad Prism 7.0 (GraphPad Software, San Diego, CA, USA), and MedCalc (MedCalc, Mariakerke, Belgium).

## Results

### Clinicopathological Parameters of HNSCC Patients

**Figure 1:**
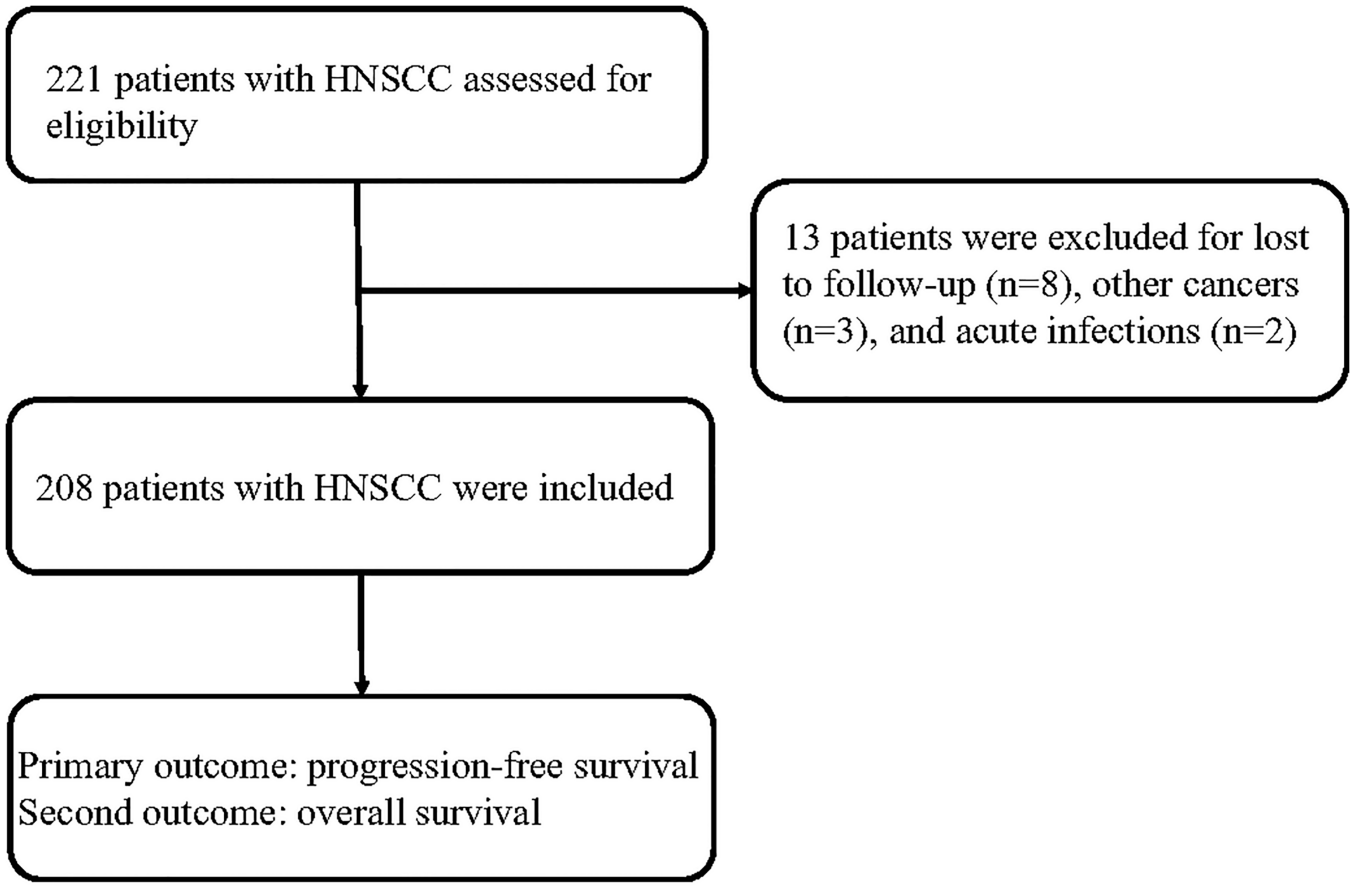
Patients’ flow diagram.

A total of 221 patients with HNSCC were assessed for inclusion in this study. Thirteen patients who were lost to follow-up (*n* = 8), had other cancers (*n* = 3), or had acute infections (*n* = 2) were excluded. Eventually, 208 patients with HNSCC were included in this study (**Figure 1**). Among these patients, 79 patients were treated in a curative intent. **Table 1** shows a summary of the relevant patient characteristics. The HNSCC patients were divided into two groups according to serum CRP level. The low-CRP group (≤ 11.3 mg/L) and the high-CRP group (> 11.3 mg/L) consisted of 122 and 86 patients, respectively. The study population consisted of 75% male patients and 25% female patients. The primary tumor locations were in the hypopharynx in 34 patients, nasopharynx in 87 patients, larynx in 72 patients, and oropharynx in 15 patients. The data indicated that HNSCC patients with higher CRP serum levels were found to exhibit more often locally advanced tumors, nodal metastases, and a higher percentage of HPV infection compared with patients with lower CRP values (**Table 1**). There were no significant differences in age, sex, smoking status, treatment protocol, or drinking status between the two groups. In addition, the serum CRP level among HNSCC patients and healthy controls was measured. We detected that the CRP level was significantly higher in patients with HNSCC than those in controls (**Supplementary Figure S1**). We also compared the CRP levels regarding treatment intention (curative group versus palliative group) and regarding M status (distant metastases) and found that that the CRP levels among the palliative group were significantly higher than those of the curative group (*P* < 0.01, **Supplementary Figure S2**). However, no significant difference was observed regarding the CRP levels among the M1 group and the M0 group (**Supplementary Figure S3**).

**Table 1:** Patients’ characteristics of HNSCC.

Variable	Number (208)	Serum CRP levels		P
		Low group (CRP, ≤11.3 mg/L)	High group (CRP, >11.3 mg/L)	
Age, years				0.477
≤60	98	60	38	
>60	110	62	48	
Sex				0.416
Male	156	94	62	
Female	52	28	24	
Smoking				0.489
Yes	142	81	61	
No	66	41	25	
Drinking				0.373
Yes	148	85	63	
No	60	37	23	
HPV infection				**0.039**
Yes	96	49	47	
No	112	73	39	
UICC stage				**0.023**
I	79	47	32	
II	63	45	18	
III	50	24	26	
IV	16	6	10	
Location				**0.005**
Hypopharynx	34	22	12	
Nasopharynx	87	60	27	
Larynx	72	36	36	
Oropharynx	15	4	11	
T stage				**0.006**
T1/T2	143	93	50	
T3/T4	65	29	36	
N stage				**0.028**
N0	150	95	55	
N1/N2/N3	58	27	31	
Treatment protocol^a^				0.104
A	16	5	11	
B	95	58	37	
C	92	55	37	
D	5	4	1	

Bold values are statistically significant (P < 0.05).

HNSCC, head and neck squamous cell carcinoma; CRP, C-reactive protein; HPV, human papillomavirus.

a Treatment protocol: A, surgery; B, radiochemotherapy; C, surgery plus postoperative radiochemotherapy; D, preoperative radiochemotherapy plus surgery.

### Association of CRP Level With Survival in Patients With HNSCC

**Figure 2:**
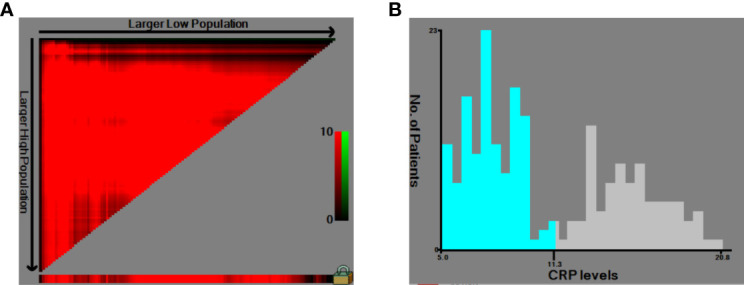
Use of X-tile analysis to identify the optimal cutoff level of the serum C-reactive protein (CRP) level. **(A)** The graph shows that the optimal cutoff value was determined by X-tile software. **(B)** Histogram was used to observe the optimal cutoff value of the CRP level.

**Figure 3:**
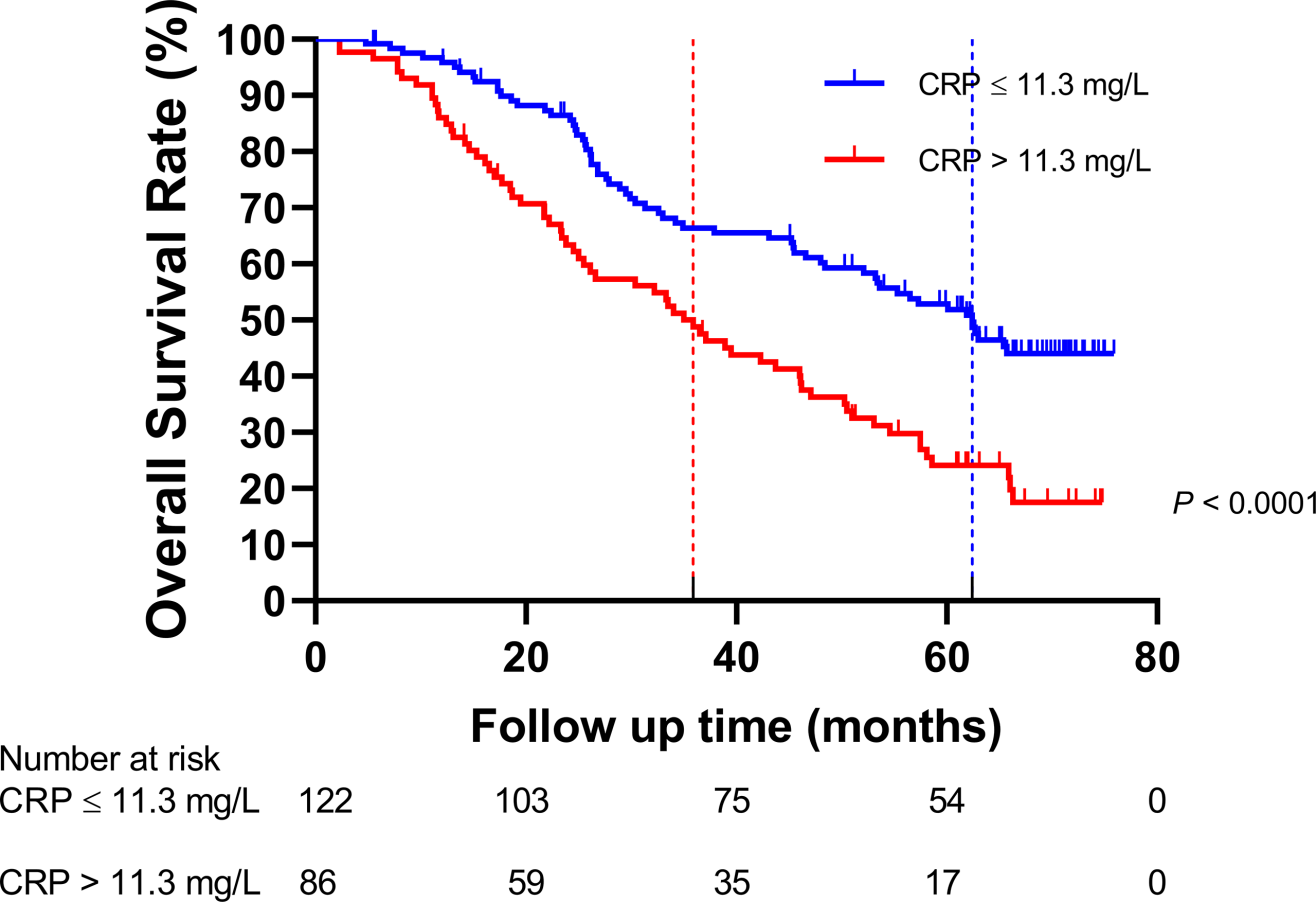
Comparison of the overall survival rate between the group with C-reactive protein (CRP) ≤11.3 mg/L and the group with CRP > 11.3 mg/L.

**Figure 4:**
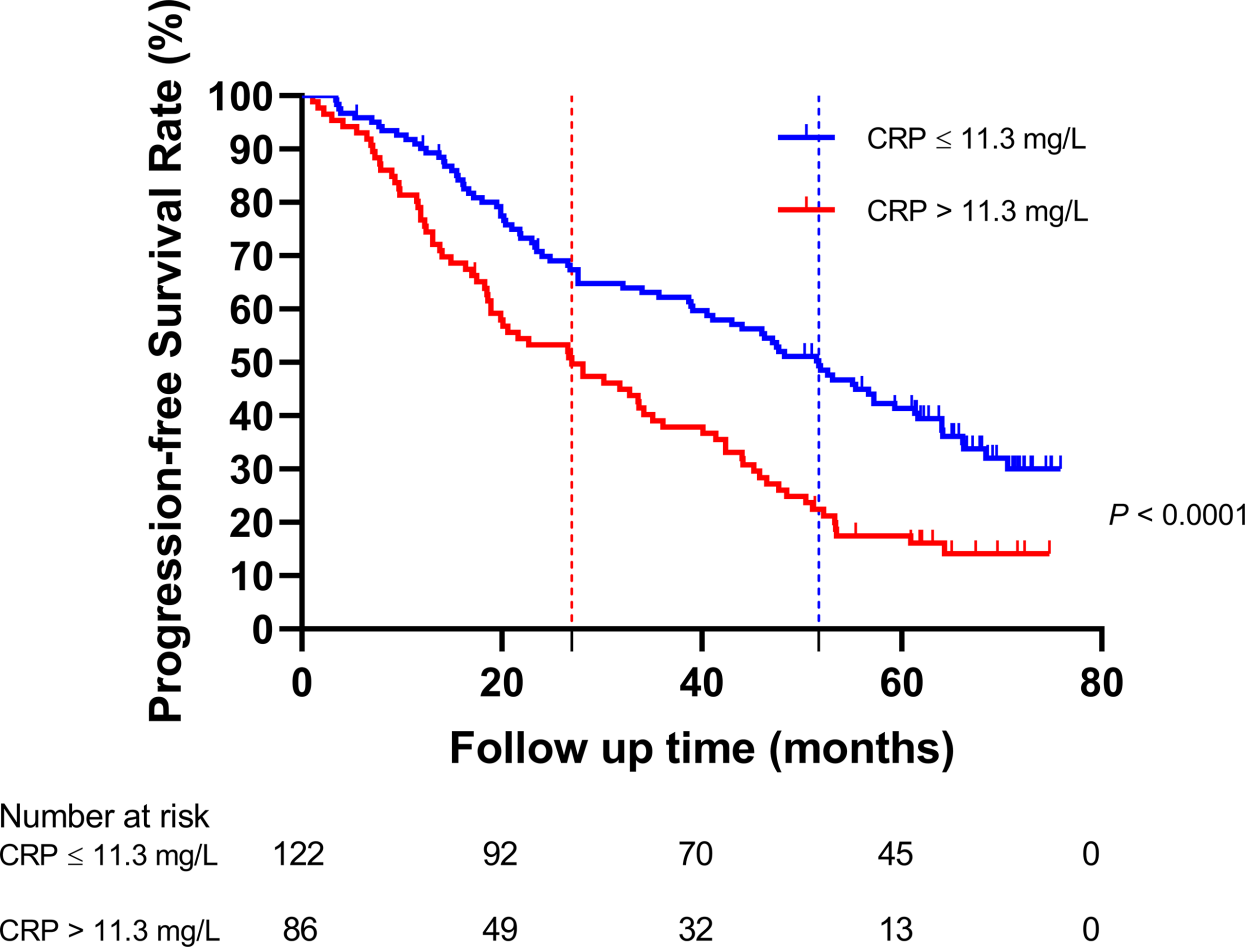
Comparison of the progression-free survival rate between the group with C-reactive protein (CRP) ≤11.3 mg/L and the group with CRP >11.3 mg/L.

The HNSCC patients were divided into two groups based on the optimal cutoff value of serum CRP level of 11.3 mg/L determined using X-tile (**Figure 2**): low-CRP group ≤11.3 mg/L and high-CRP group >11.3 mg/L. Kaplan–Meier analysis and log-rank test were used to evaluate the prognostic value of CRP. The median OS among the CRP-low and CRP-high groups was 62.4 and 35.9 months, respectively. The median PFS among the CRP-low and CRP-high groups was 51.7 and 27.0 months, respectively. The 1-year OS rates among the CRP-low and CRP-high groups were 96.72 and 86.05%, respectively. The 2-year OS rates among the CRP-low and CRP-high groups were 86.89 and 63.95%, respectively. The 1-year PFS rates among the CRP-low and CRP-high groups were 90.16 and 76.74%, respectively. The 2-year PFS rates among the CRP-low and CRP-high groups were 71.31 and 53.49%, respectively. Data indicated that patients with HNSCC in the low-CRP group showed significantly better survival in terms of OS (*P* < 0.0001, **Figure 3**) and PFS (*P* < 0.0001, **Figure 4**) than those in the high-CRP group. To sum up, these findings suggested that serum CRP level was associated with the survival of patients with HNSCC.

### Prognostic Markers for OS and PFS in Patients With HNSCC

Univariate and multivariate Cox analyses were conducted to identify the prognostic markers for patients with HNSCC. The univariate analysis revealed that older age (>60 years), smoking, N1–3 stage, T3–4 stage, UICC III stage, UICC IV stage, and serum CRP level >11.3 mg/L were significantly associated with a poorer OS (**Table 2**). The multivariate analysis suggested that N1–3 stage (HR, 4.98; 95% CI, 2.04–12.17, *P* < 0.001), UICC II stage (HR, 1.65; 95% CI, 1.01–2.71, *P* = 0.048), and serum CRP level >11.3 mg/L (HR, 1.90; 95% CI, 1.32–2.73, *P* = 0.001) were independent prognostic factors for OS. The univariate analysis revealed that older age (>60 years), smoking, N1–3 stage, T3–4 stage, UICC III stage, UICC IV stage, and serum CRP level >11.3 mg/L were also negatively associated with PFS (**Table 3**). By multivariate analysis with enter method, the data showed that N1–3 stage (HR, 3.23; 95% CI, 1.44–7.25, *P* = 0.004) and serum CRP level >11.3 mg/L (HR, 1.75; 95% CI, 1.25–2.45, *P* = 0.001) were shown to be independent prognostic markers for PFS. It is of note that CRP remained a prognostic parameter for OS and PFS in patients with HNSCC even if it was entered as a continuous variable. Taken together, N1–3 stage and serum CRP level >11.3 mg/L were shown to be independent prognostic markers for patients with HNSCC.

**Table 2:** Univariate and multivariate analysis of prognostic factors affecting the overall survival in patients with HNSCC.

Variable	Category	Univariate	*P*	Multivariate	P
		HR (95% CI)		HR (95% CI)	
Age	> 60 versus ≤ 60	**2.35** (**1.63**–**3.40**)	<**0.001**	1.29 (0.85–1.96)	0.233
Sex	Male versus female	1.54 (0.99–2.39)	0.053		
Smoking	Yes versus no	**1.50** (**1.01**–**2.23**)	**0.042**	1.24 (0.83–1.86)	0.299
Drinking	Yes versus no	1.28 (0.86–1.89)	0.223		
HPV infection	Yes versus no	1.21 (0.85–1.71)	0.291		
UICC stage	II versus I	1.61 (0.98–2.64)	0.060	**1.65** (**1.01**–**2.71**)	**0.048**
	III versus I	**13.83** (**8.25**–**23.18**)	<**0.001**	0.58 (0.06–5.29)	0.628
	IV versus I	**10.82** (**5.68**–**20.64**)	<**0.001**	0.58 (0.06–5.37)	0.630
T stage	T3–4 versus T1–2	**11.31** (**7.35**–**17.41**)	<**0.001**	6.15 (0.82–46.34)	0.078
N stage	N1–3 versus N0	**12.17** (**7.93**–**18.67**)	<**0.001**	**4.98** (**2.04**–**12.17**)	<**0.001**
Treatment protocol^a^	B versus A	0.95(0.48–1.86)	0.878		
	C versus A	1.07(0.54–2.09)	0.852		
	D versus A	1.68(0.53–5.37)	0.381		
CRP levels (mg/L)	>11.3 *versus ≤*11.3	**2.08** (**1.46**–**2.95**)	<**0.001**	**1.90** (**1.32**–**2.73**)	**0.001**

Bold values are statistically significant (P < 0.05).

HNSCC, head and neck squamous cell carcinoma; CRP, C-reactive protein; HPV, human papillomavirus.

a Treatment protocol: A, surgery; B, radiochemotherapy; C, surgery and radiochemotherapy; D, preoperative radiochemotherapy and surgery.

**Table 3:** Univariate and multivariate analysis of prognostic factors affecting the progression-free survival in patients with HNSCC.

Variable	Category	Univariate	*P*	Multivariate	P
		HR (95% CI)		HR (95% CI)	
Age	>60 *versus ≤*60	**2.09** (**1.50**–**2.91**)	<**0.001**	1.20 (0.82–1.75)	0.354
Sex	Male versus female	1.36 (0.92–2.00)	0.122		
Smoking	Yes versus no	**1.53** (**1.07**–**2.20**)	**0.022**	1.30 (0.89–1.88)	0.175
Drinking	Yes versus no	1.33 (0.92–1.91)	0.130		
HPV infection	Yes versus no	1.13 (0.82–1.56)	0.456		
UICC stage	II versus I	1.42 (0.92–2.17)	0.113	1.44 (0.94–2.21)	0.098
	III versus I	**10.98** (**6.88**–**17.54**)	<**0.001**	0.59 (0.07–5.10)	0.628
	IV versus I	**10.82** (**5.68**–**20.64**)	<**0.001**	0.57 (0.07–4.99)	0.610
T stage	T3–4 versus T1–2	**9.92** (**6.60**–**14.90**)	<**0.001**	7.10 (0.95–53.23)	0.057
N stage	N1–3 versus N0	**9.30** (**6.23**–**13.86**)	<**0.001**	**3.23** (**1.44**–**7.25**)	**0.004**
Treatment protocol^a^	B versus A	1.19 (0.61–2.32)	0.604		
	C versus A	1.31 (0.68–2.55)	0.852		
	D versus A	2.29 (0.78–6.73)	0.130		
CRP levels (mg/L)	>11.3 versus ≤11.3	**1.94** (**1.40**–**2.68**)	<**0.001**	**1.75** (**1.25**–**2.45**)	**0.001**

Bold values are statistically significant (P < 0.05).

HNSCC, head and neck squamous cell carcinoma; CRP, C-reactive protein; HPV, human papillomavirus.

a Treatment protocol: A, surgery; B, radiochemotherapy; C, surgery and radiochemotherapy; D, preoperative radiochemotherapy and surgery.

## Discussion

In this study, we found that serum CRP level was associated with UICC stage, tumor localization, T stage, N stage, and HPV infection. In addition, N1–3 stage and a high serum CRP level (>11.3 mg/L) were shown to be associated with poorer OS and PFS in patients with HNSCC.

Salas et al. first explored the association between CRP level and survival of patients with unresectable HNSCC and found that CRP influenced the OS of HNSCC on univariate analysis, but not on multivariate analysis (25). However, they reported that, among all biological nutritional factors, CRP level correlated with the response to radiochemotherapy (25). Peter et al. suggested that a higher CRP level was a negative prognostic factor for OS in HNSCC patients in Germany (23). A single-center analysis of 246 elderly (≥65 years) HNSCC patients undergoing (chemo)radiotherapy could show that CRP levels were a prognostic parameter in the univariate Cox analysis, but not in the multivariate analysis (24). However, a subsequent bi-center study, in which a survival score for elderly HNSCC patients was developed and validated, demonstrated that CRP levels were an independent prognosticator for OS in elderly HNSCC patients (31). Magnes et al. reported that a higher CRP level was associated with OS in Australian patients with recurrent or metastatic HNSCC treated with cetuximab and chemotherapy (22). A Canadian study revealed that serum CRP level was an independent predictor of relapse-free survival and OS in advanced-stage HNSCC despite p16-positive status (27). Two Asian studies from Korea and Japan yielded inconsistent findings (19, 28); the Korean study showed that CRP level was not associated with the survival of HNSCC patients treated with chemoradiotherapy (19, 28), while the Japanese study indicated that a low CRP level was significantly associated with better OS in patients with recurrent and/or metastatic HNSCC receiving salvage chemotherapy following treatment with nivolumab (28).

With regard to oral cancer, Khandavilli et al. found that preoperative CRP level was associated with OS in patients with oral squamous cell carcinoma (OSCC) treated with primary surgery (18), but this was not observed in other studies (14, 15). Kruse et al. showed that there were no correlations between preoperative CRP level and either recurrence or metastasis of oral cancer (21). In addition, a high pretreatment CRP level was a consistent prognostic indicator of poor OS and cancer-specific survival (CSS) in patients with orohypopharyngeal squamous cell carcinoma (17, 20). In an Australian study of tongue squamous cell carcinoma (TSCC), Graupp et al. reported that the CRP level was related to the OS, but not DFS, in TSCC (16). Two Chinese studies indicated that serum CRP level predicted a poor prognosis in patients with nasopharyngeal carcinoma (26, 29). Some other groups shed light on the effects of the combination of CRP and other markers on the survival of HNSCC patients. Two Taiwanese studies observed that a squamous cell carcinoma antigen level of 2.0 ng/ml and a CRP level of 5.0 mg/L were associated with the prognosis of OSCC (32) and pharyngolaryngeal carcinoma (33). Ko et al. reported that the preoperative CRP–lymphocyte ratio was related to the prognosis of patients with OSCC (34). The CRP/albumin ratio could predict poor survival in patients with hypopharyngeal and laryngeal cancer (35).

Due to the discrepancies in the results of the studies outlined (**Supplementary Table 1**) above, Chen et al. conducted a meta-analysis and reported that an elevated pretreatment CRP level is an indicator of poor prognosis in HNSCC (36). To date, no Chinese studies have investigated the effects of serum CRP level on the prognosis of patients with HNSCC, including cancers of the hypopharynx, nasopharynx, larynx, and oropharynx. Therefore, we designed this cohort study and found that a high serum CRP level was associated with poorer OS and PFS in Han Chinese HNSCC patients. There are several potential explanations for the partial inconsistencies of our findings with those of previous studies (22–25, 27, 28). One, clinical heterogeneity, including cancer location, tumor stage, and treatment method, may have affected the final results. Two, the primary outcomes, including OS, DFS, and CSS, were different between studies. Three, the cutoff value for the serum CRP level differed between studies, which might result in conflicting results. Four, the sample sizes varied between studies; studies with a limited sample size may yield false-negative results. Lastly, there were differences in the ethnicity of the study populations between studies.

Notably, the PFS of patients with HNSCC was only investigated in one previous study in South Korea by Kim et al. (19). They found that CRP level was not an independent predictor of PFS and tumor recurrence on multivariate analysis (19). In this study, we observed that a high CRP level (>11.3 mg/L) was an unfavorable prognostic factor for PFS in patients with HNSCC, which was inconsistent with the findings of Kim et al. (19). To our knowledge, this is the first study to demonstrate an association of high CRP level (>11.3 mg/L) with poorer PFS in Han Chinese patients with HNSCC.

The mechanisms underlying the association between CRP level and survival of HNSCC patients are unclear, but the following factors may play roles. Inflammation promotes the development of cancer and all stages of tumorigenesis (37). Inflammation exerts a major effect on the composition of the tumor microenvironment, particularly on the plasticity of both stromal and tumor cells (38). Inflammation is an intricate process involving dozens of molecules, including interleukin 6 (IL-6) and CRP (39). CRP, an acute-phase inflammatory protein, is synthesized in the liver in response to inflammation. Many studies have demonstrated that CRP was associated with cancer survival (36, 40–45). In addition, the production of CRP in the liver is stimulated by IL-6 (46). IL-6 accelerates angiogenesis, which promotes the progression and metastasis of tumors (47). Cancer cells could, in turn, produce several chemokines and cytokines, thus increasing the serum CRP level (48). In addition, CRP may mediate carcinogenesis and cancer progression *via* activation of the FcgRs/MAPK/ERK, FcgRs/NF-κB/NLRP3, and FcgRs/IL-6/AKT/STAT3 pathways (12). She et al. found that the network of CRP-interacting proteins may activate the PI3K/Akt signaling pathway, thereby contributing to the pathogenesis of hepatocellular carcinoma (49).

Several limitations were shown in this study. First, the study population was relatively small. Therefore, additional studies with larger sample sizes in China are required. Second, the clinical heterogeneity of HNSCC was inevitable (e.g., diverse treatment strategies were applied in the patients). Third, other pivotal factors affecting the prognosis of HNSCC were not explored in this study. Fourth, as this was a retrospective study of the effects of serum CRP level on survival in HNSCC patients at a single center, not all laboratory indexes were available for all HNSCC patients. Prospective studies at multiple centers should therefore be designed in the future. Lastly, a further study is needed to determine the mechanisms underlying the results of this study, which will help to identify new targets for innovative biological therapy in HNSCC patients.

## Conclusion

In total, this study shows that a higher CRP level is associated with poorer PFS and OS in patients with HNSCC. Therefore, CRP level may be useful as a prognostic indicator for HNSCC patients. Serum CRP level can be measured simply and repeatedly and therefore could be regarded as a routine clinical marker in patients with HNSCC.

## Data Availability Statement

The original contributions presented in the study are included in the article/**Supplementary Material**. Further inquiries can be directed to the corresponding author.

## Ethics Statement

The studies involving human participants were reviewed and approved by Huaian No. 1 People’s Hospital. The patients/participants provided their written informed consent to participate in this study.

## Author Contributions

Conception and design: YZ and DG. Administrative support: DG. Provision of study materials or patients: YZ and DG. Collection and assembly of data: YZ. Data analysis and interpretation: YZ and DG. Manuscript writing: YZ and DG. All authors contributed to the article and approved the submitted version.

## Conflict of Interest

The authors declare that the research was conducted in the absence of any commercial or financial relationships that could be construed as a potential conflict of interest.

## Publisher’s Note

All claims expressed in this article are solely those of the authors and do not necessarily represent those of their affiliated organizations, or those of the publisher, the editors and the reviewers. Any product that may be evaluated in this article, or claim that may be made by its manufacturer, is not guaranteed or endorsed by the publisher.

## Supplementary Material

Supplementary Figure 1 Comparison of the C-reactive protein levels between patients with head and neck squamous cell carcinoma and the controls.

Supplementary Figure 2 Comparison of the C-reactive protein levels between the palliative group and the curative group.

Supplementary Figure 3 Comparison of the C-reactive protein levels between the M1 group and the M0 group.
