# Supplementary figures and images for: Prognostic Impact of Serum CRP Level in Head and Neck Squamous Cell Carcinoma
Source: Front Oncol. 2022 Jun 29;12:889844. doi: 10.3389/fonc.2022.889844 (PMC9277075; doi:10.3389/fonc.2022.889844)

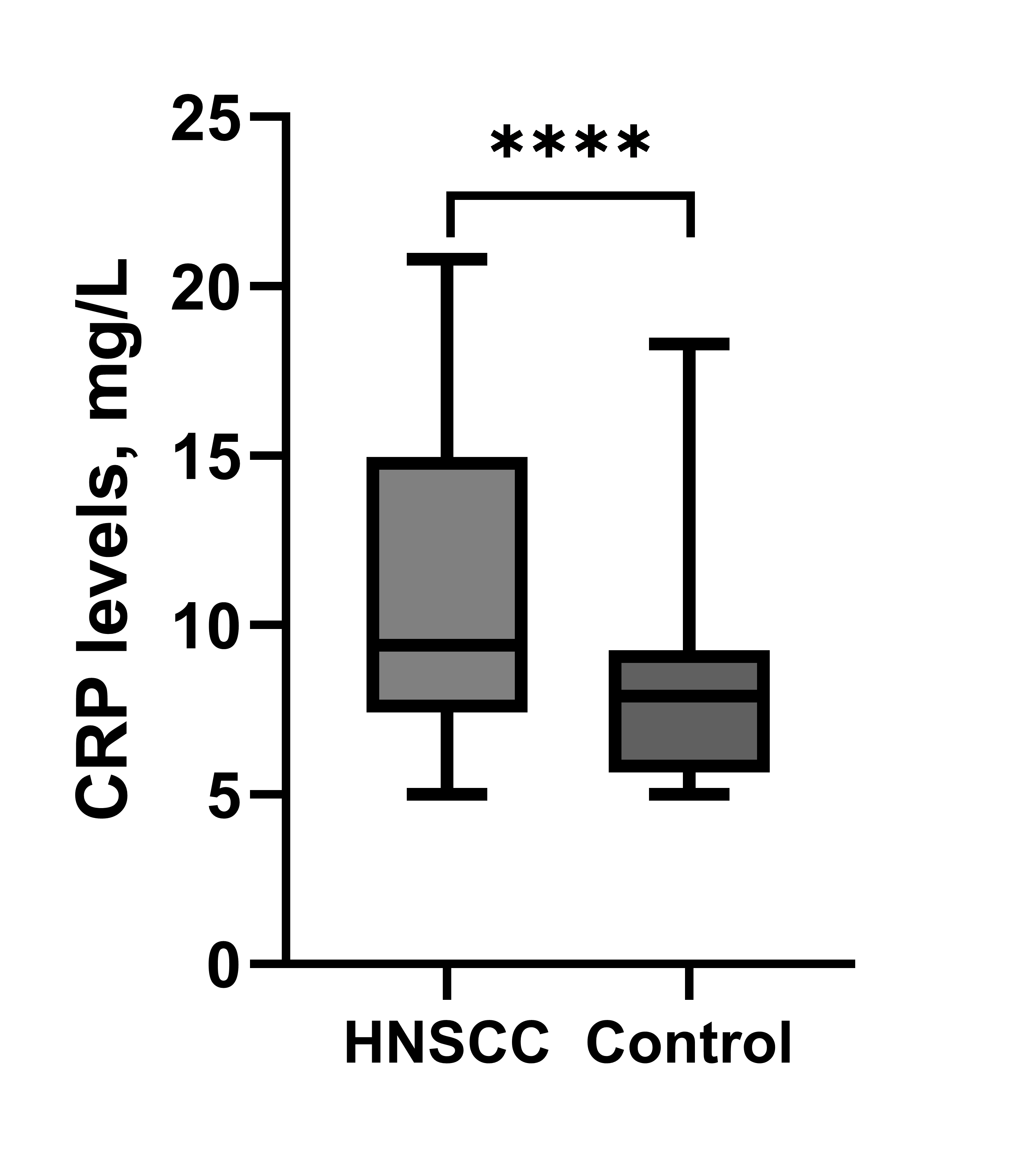

Supplement: Supplementary Figure 1 — Comparison of the C-reactive protein levels between patients with head and neck squamous cell carcinoma and the controls. [file Image_1.tif]

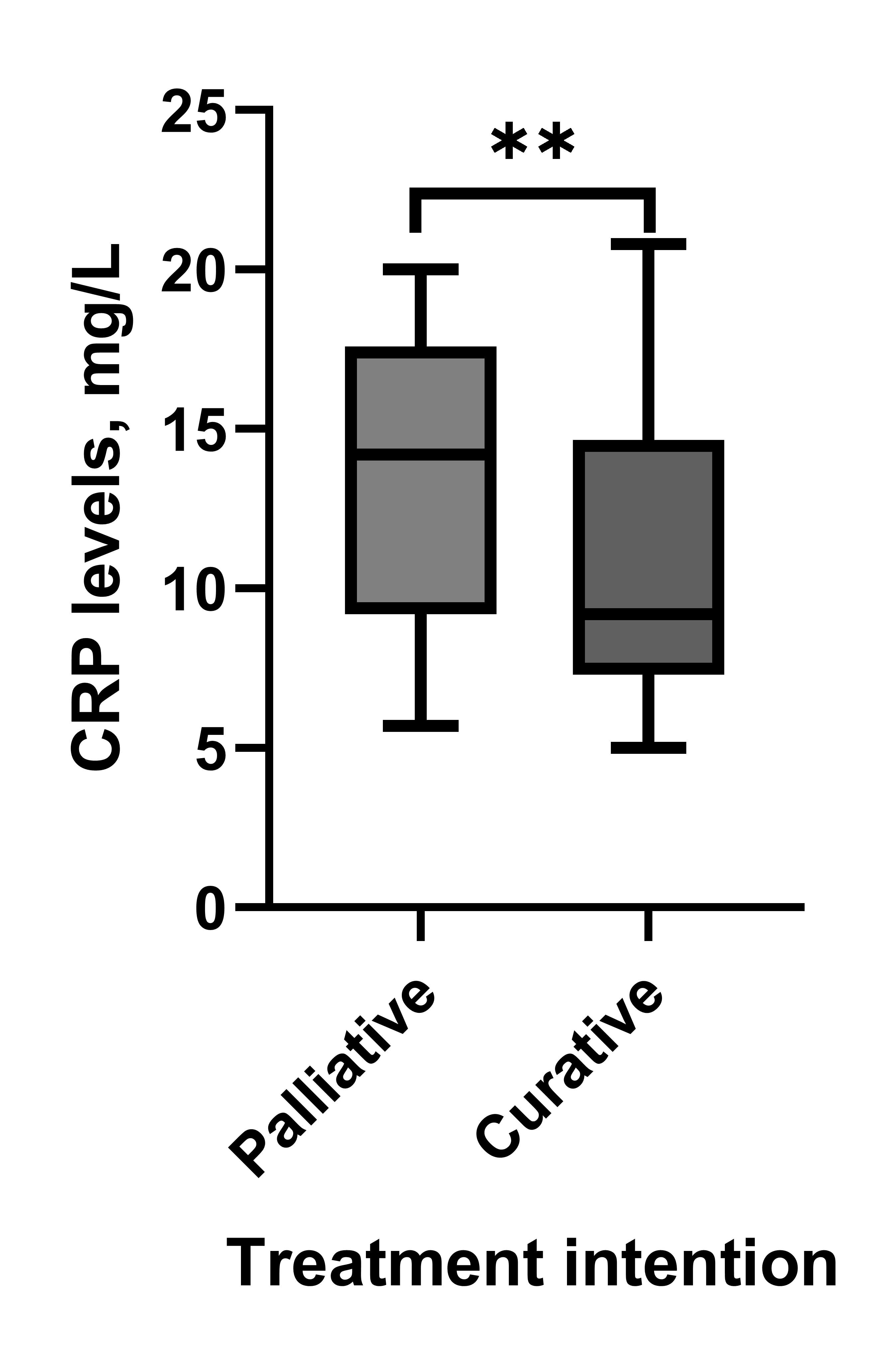

Supplement: Supplementary Figure 2 — Comparison of the C-reactive protein levels between the palliative group and the curative group. [file Image_2.tif]

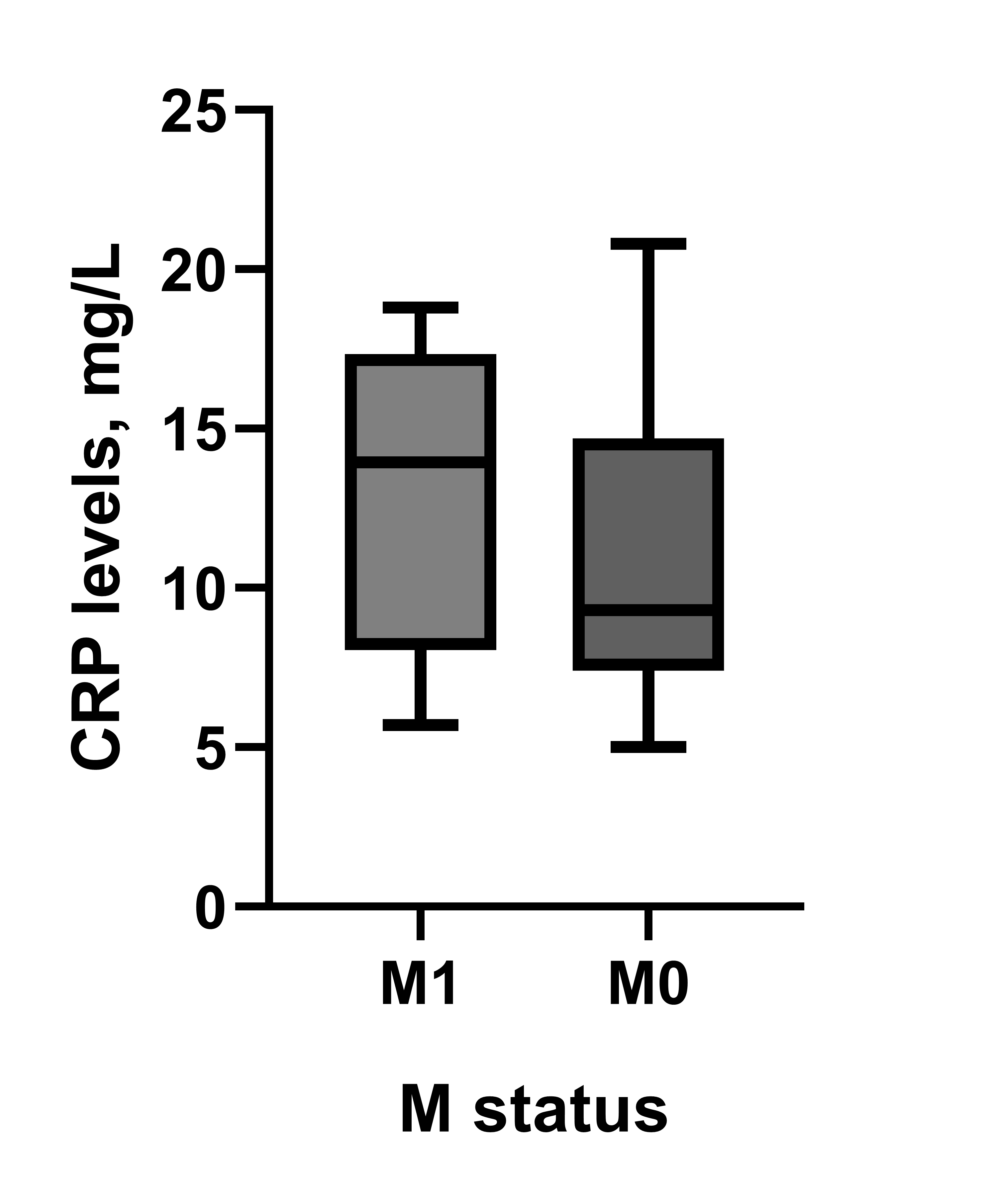

Supplement: Supplementary Figure 3 — Comparison of the C-reactive protein levels between the M1 group and the M0 group. [file Image_3.tif]
