# Supplementary material for: Prognostic Impact of Serum CRP Level in Head and Neck Squamous Cell Carcinoma
Source: Front Oncol. 2022 Jun 29;12:889844. doi: 10.3389/fonc.2022.889844 (PMC9277075; doi:10.3389/fonc.2022.889844)
Supplement: Supplementary file 4 [file Table_1.docx]

**Supplementary Table 1** The association with CRP levels and the survival of HNSCC in eligible studies.

| **Author and year** | **Cut-off value (mg/L)** | **Association with survival of HNSCC^#^** |  |
| --- | --- | --- | --- |
| Salas et al, 2008 | NA | Yes |  |
| Khandavilli et al, 2009 | 5 | No | |
| Kruse et al, 2010 | 5 | Yes | |
| Peter et al, 2012 | 2 | Yes | |
| Tang et al, 2015 | 1.96 | Yes |  |
| Zeng et al, 2015 | 8 | Yes |  |
| Kim et al, 2016 | NA | No |  |
| Eder-Czembirek et al, 2016 | 10 | No |  |
| Katano et al, 2017 | 3 | Yes |  |
| Magnes et al, 2017 | 8.5 | Yes |  |
| Kawakita et al, 2017 | 3.9 | Yes |  |
| Graupp et al, 2018 | 5 | Yes |  |
| DE Paz et al, 2019 | 5 | Yes |  |
| Rühle et al, 2020 | 5 | No |  |
| Valdes et al, 2020 | 6 | Yes |  |
| Wakasaki et al, 2020 | 1.2 | Yes |  |
| Knittelfelder et al, 2020 | 5 | Yes |  |
| Ruhle et al, 2021 | 5 | Yes |  |

NA, not available; #Yes means that CRP was associated with the cancer-specific survival or progress-free survival or recurrence-free survival or overall survival; No means that CRP was associated with the survival of HNSCC.
